# Advances in high-throughput mutation breeding systems integrating atmospheric and room-temperature plasma (ARTP) with droplet-based microfluidics

**DOI:** 10.1186/s12934-025-02873-z

**Published:** 2025-12-30

**Authors:** Lijuan Xu, Mengxin Guo, Dahai Jiang, Jianchun Jiang, Liming Lu

**Affiliations:** 1 Academy of Advanced Carbon Conversion Technology, Huaqiao University, Xiamen, China; 2 Fujian Provincial Key Laboratory of Biomass Low-Carbon Conversion, Huaqiao University, Xiamen, China; 3 College of Chemical Engineering, Huaqiao University, Xiamen, China; 4 Institute of Chemical Industry of Forest Products, Nanjing, China

**Keywords:** Atmospheric and room-temperature plasma (ARTP), Droplet-based microfluidic technology, Microorganisms, High-throughput mutagenesis and screening system

## Abstract

In recent years, the integration of atmospheric and room-temperature plasma (ARTP) mutagenesis with droplet-based microfluidic (DBMF) technology has enabled the development of a novel high-efficiency mutagenesis and screening system. This system not only enhances microbial mutagenesis efficiency but also achieves precise screening and high-throughput detection, demonstrating broad applications in biosynthesis, fermentation engineering, biological feed production, edible fungus breeding, and environmental remediation. This review comprehensively elaborates on the principles and advantages of the system and discusses its diverse applications across multiple fields.

## Introduction

Microbial breeding plays a pivotal role in fermentation engineering, pharmaceuticals, environmental protection, and other domains. In fermentation engineering, efficient microbial breeding is critical for improving production efficiency, product quality, and cost reduction in food, beverages, pharmaceuticals, and biofuels. In pharmaceuticals, microbial metabolites serve as essential sources of therapeutic agents, while in environmental protection, microorganisms are utilized for wastewater treatment and pollutant degradation. Conventional mutagenesis and breeding methods-such as chemical mutagenesis, radiation mutagenesis, and transgenic techniques-face limitations: chemical mutagenesis suffers from unpredictable mutations due to reagent specificity and concentration constraints; radiation mutagenesis often compromises cell viability under high lethality; and transgenic technology, despite enabling targeted trait enhancement, encounters regulatory and public acceptance challenges. Consequently, the development of advanced mutagenesis and screening systems has become imperative. Given that atmospheric and room-temperature plasma (ARTP) mutagenesis technology enables rapid and efficient induction of microbial mutations under ambient conditions, its integration with droplet-based microfluidics (DBMF) technology facilitates high-throughput screening and accelerated strain improvement. This combination thereby offers pioneering opportunities for microbial enhancement and applications.

## Principles and advantages of the system

### System principles

#### Atmospheric and Room-Temperature plasma (ARTP) mutagenesis

**Fig. 1:**
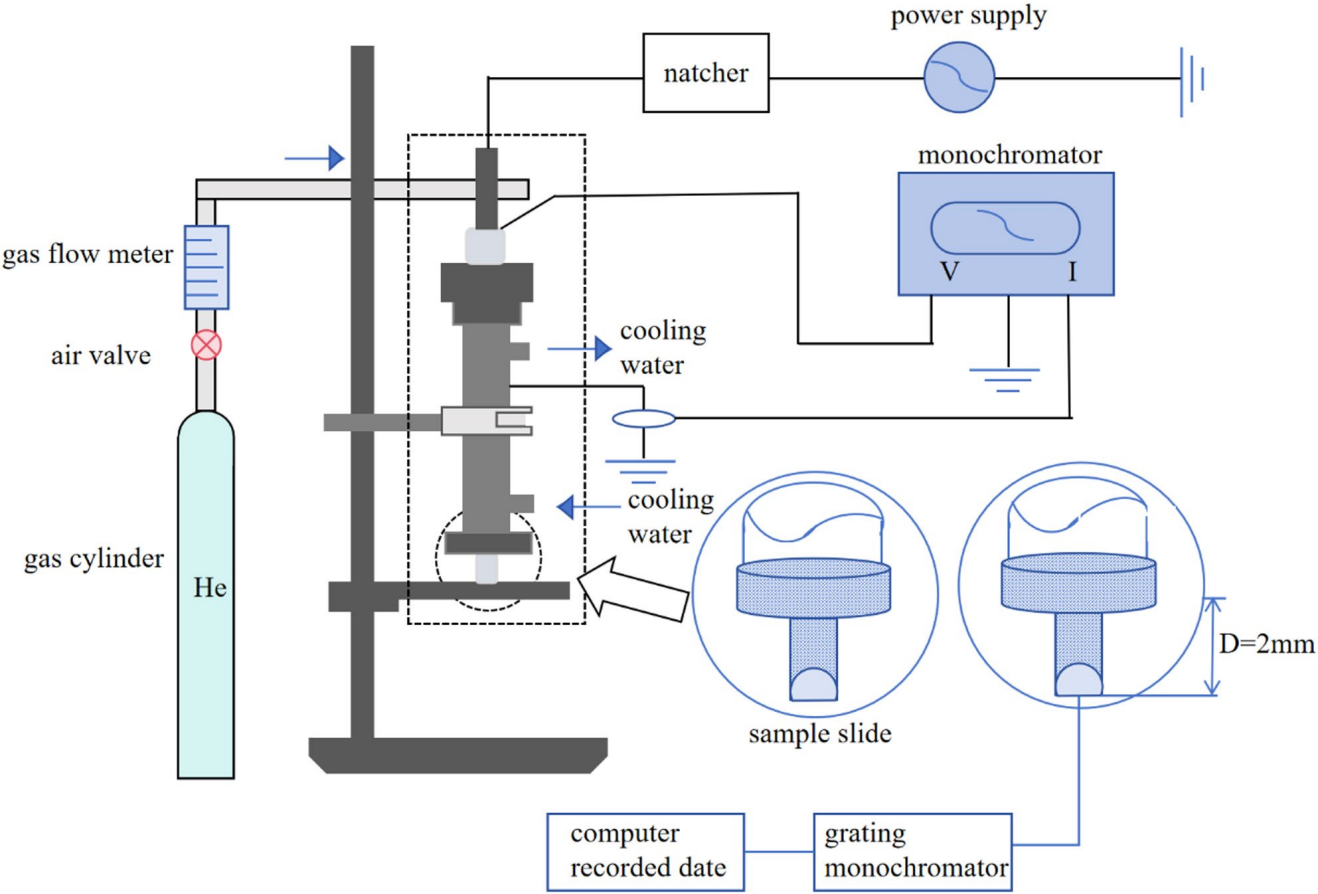
Schematic diagram of the Atmospheric and Room Temperature Plasma (ARTP) generation device

ARTP (Atmospheric Room Temperature Plasma, ARTP) is a new radio frequency discharge technology, the working principle is that when the inert gas flows through the two bare metal electrodes, under the influence of an applied radio-frequency electric field, electrons gain energy and undergo both elastic and inelastic collisions with neutral gas molecules. This energy exchange leads to gas ionization, resulting in plasma formation with a specific degree of ionization. The composition of active particles within the plasma varies depending on the applied voltage and the type of gas source used (ARTP plasma generator device schematic diagram in Fig. 1). Studies have shown that the active particles can be effectively used in the genetic material of the cell and lead to DNA structural damage [1], and then utilize the cell’s own high fault-tolerant repair mechanism to generate a large number of mutation sites, and ultimately obtain a large-capacity gene mutation library.

The atmospheric pressure room temperature plasma source using helium as the working gas contains a variety of chemically active particle components such as OH, nitrogen molecule di-positive system, nitrogen molecule one-negative system, excited helium atoms, hydrogen atoms, and oxygen atoms [2], etc. The active energy-enriched particles of the ARTP cause damages to the genetic material of the bacterial strains/plants/cells, etc., and induce the biological The SOS repair process is a highly error-tolerant repair process [3], which generates a rich variety of mismatch sites and ultimately stabilizes the genetic inheritance to form mutant strains, and the intensity of SOS repair is highly correlated with the degree of DNA damage [2]. ARTP has a strong effect on the genetic material of eukaryotic organisms, and thus shows a more efficient mutation performance and a wider range of application than other mutagenesis methods. After genome sequencing, it is known that the mutant strains obtained by ARTP mutation treatment have more abundant mutation sites [4]. ARTP has been widely used in industrial microbial breeding and achieved good results due to the advantages of mild operating conditions, rich variety of active particles, controllable conditions, and safety and stability.

#### Droplet-based microfluidic (DBMF) technology

Droplet-based microfluidics (DBMF), as an advanced branch of microfluidic technology, represents a breakthrough beyond traditional continuous-flow systems. Its core principle involves precisely manipulating two immiscible fluid phases to generate thousands to millions of discrete microdroplet units (10–200 μm in diameter) in situ within microscale channels. Each droplet functions as an independent, compartmentalized microreactor, enabling the encapsulation of single molecules or single cells-including proteins, nucleic acids, and whole cells [5]. Leveraging the synergistic advantages of miniaturization (picoliter-scale volumes), parallelization (>20,000 droplets/second throughput), and compartmentalization (physical isolation by oil phase to prevent cross-contamination), DBMF overcomes the limitations of macroscale reactors in detection sensitivity, reagent consumption, and processing efficiency. This technology is now widely adopted for single-cell omics analysis, high-throughput drug screening, and rapid pathogen detection.

Within microbial engineering, its sealed microenvironment constitutes a ​promising platform​ for strain engineering, offering distinct advantages for high-throughput screening: Researchers can achieve massively parallel microbial cultivation to accelerate phenotypic characterization [6]; dynamic stress acclimation to enhance strain robustness [7]; and ultrahigh-throughput enzyme activity screening using fluorescence labeling to precisely isolate rare mutants exhibiting >20fold performance improvements [8]– [9].

**Fig. 2:**
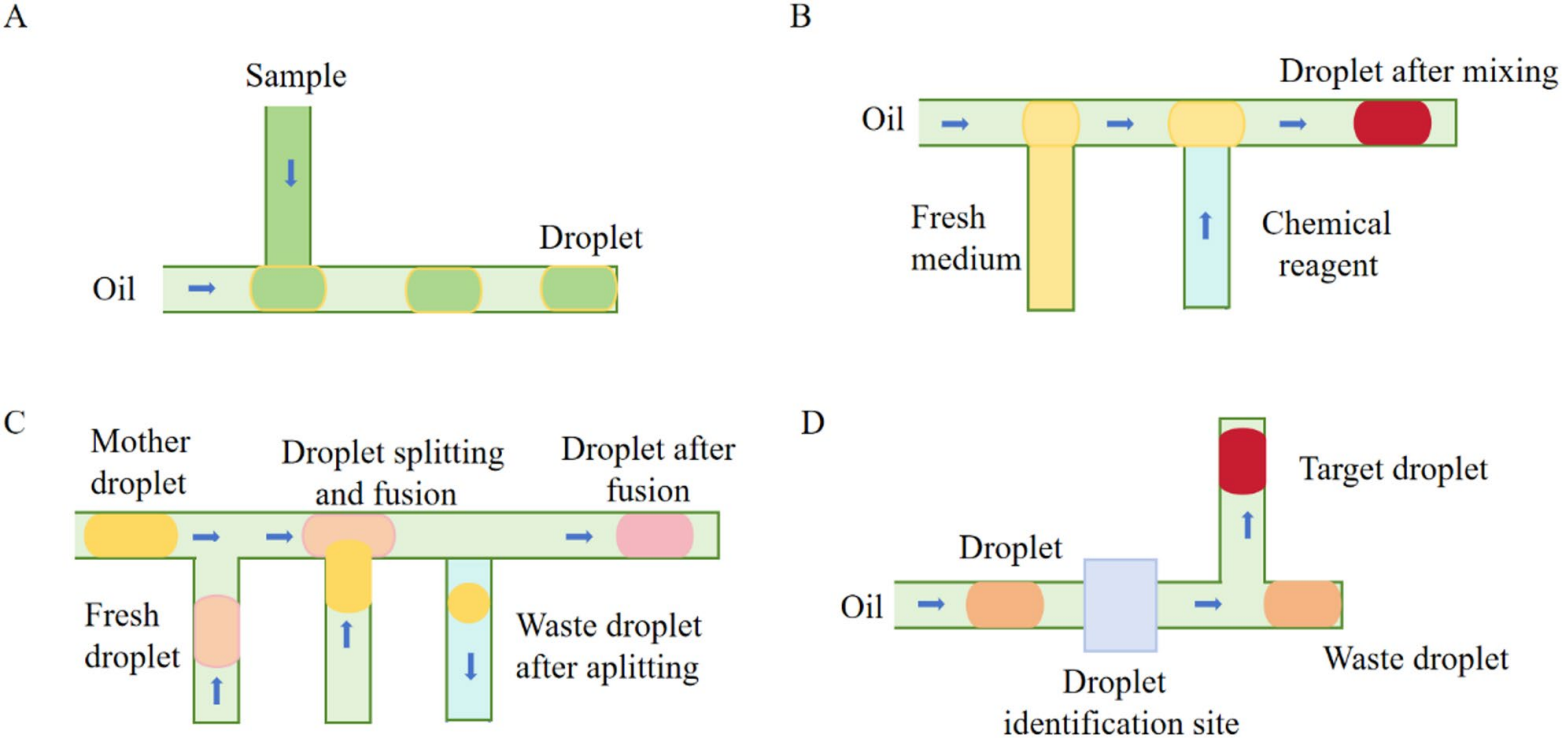
Droplet Manipulation System (A: Droplet generation; B: Multi-concentration gradient droplet generation; C: Droplet splitting and fusion; D: Droplet sorting)

DBMF for microbial research has these features:

1. Microorganisms are encapsulated in droplets, isolating a few or even single cells. This eliminates the effects of growth rate differences and interspecies competition [10], aiding the study of rare and slow-growing microbes in complex samples. The rapid accumulation of metabolites in droplets helps activate concentration-dependent physiological pathways like quorum sensing [11].
2. Microfluidic devices can generate highly uniform droplets at up to 20,000 Hz and perform high-throughput analysis, enabling ultra-high-throughput microbial identification and screening [12]– [13].
3. In DBMF systems, channels can be custom-designed and various control modules integrated to precisely manipulate droplets. This includes injection, mixing, dispersion, long-term incubation, and sorting [14]. This allows rapid and precise introduction of multiple detection reagents and stimulants into microbial cells [15]– [16], creating diverse and controllable environments for high-throughput and precise microbial cell manipulation [17] .
4. The main components of microbial DBMF technology are: Droplet generation, including generating single-phase dispersed droplets, droplet arrays, and droplets with different material surfaces or internal components [18] ; Manipulating droplets and their internal components [19] ; Analyzing microorganisms within droplets [20]. (See Fig. 2 for the composition of the droplet operation system.)

### Integrated ARTP-DBMF system for accelerated microbial strain development

**Fig. 3:**
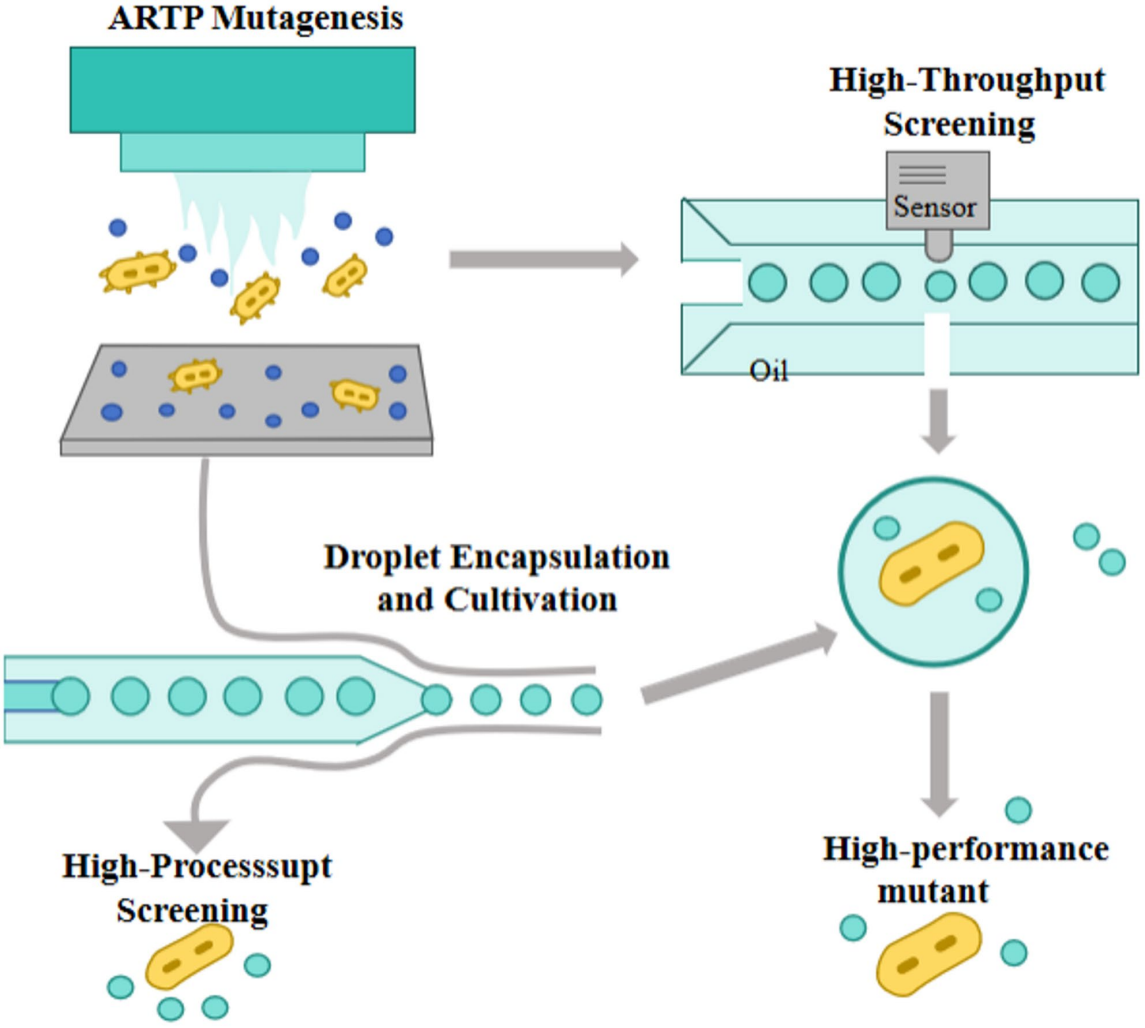
Integrated ARTP-DBMF System

The integration of Atmospheric and Room-Temperature Plasma (ARTP) mutagenesis with Droplet-Based Microfluidics (DBMF) establishes a next-generation closed-loop platform for microbial strain development (Fig. 3). This combination bridges a long-standing gap between random mutagenesis and high-resolution screening, providing a unified workflow that accelerates mutation–selection cycles by several orders of magnitude compared to conventional methods. This system operates through three functionally coupled phases, creating a powerful feedback loop that dramatically accelerates the ‘mutagenize-screen-learn’ cycle.

#### Mutagenesis via ARTP

ARTP exposes microbial cells to reactive plasma species under atmospheric pressure, inducing extensive genomic mutations through non-contact and mild treatment. This process generates a genetically diverse mutant pool while maintaining high cell viability-an essential prerequisite for downstream microfluidic encapsulation. The random yet controllable mutation profile of ARTP creates the genetic foundation required for large-scale, high-throughput screening in microdroplet systems.

#### Droplet encapsulation and cultivation

DBMF, as an advanced microfluidic branch, manipulates two immiscible fluid phases to produce thousands to millions of picoliter-scale droplets (10–200 μm) within microscale channels. Each droplet functions as an independent, compartmentalized microreactor capable of encapsulating single cells or biomolecules. Compared with macroscopic reactors, DBMF achieves extreme miniaturization, parallelization (> 20,000 droplets s^− 1^), and isolation, which drastically reduce reagent consumption (to 10^− 6^-10^− 7^fold) and prevent cross-contamination. Within this confined environment, ARTP-induced mutants can be cultivated, stressed, or induced under precisely controlled conditions, enabling dynamic phenotypic responses and accelerated adaptive evolution.

#### High-throughput screening

During incubation, droplets are dynamically monitored for target phenotypes using fluorescence probes or absorbance sensors integrated into the microfluidic chip. Active sorting mechanisms then isolate high-performance mutants at rates exceeding 20,000 droplets per hour-10^4^-10^5^ times faster than microplate-based assays.

The integration leverages ARTP’s ​​random mutagenesis breadth​​ and DBMF’s ​​single-cell resolution screening​​. ARTP enriches genetic diversity, while DBMF enables ultra-high-throughput phenotypic analysis under controlled microenvironments. This closed-loop system-from mutation induction to precision screening-dramatically accelerates strain improvement cycles compared to conventional methods.

### System advantages

The ARTP-DBMF mutation breeding system demonstrates a synergistic convergence of broad-spectrum mutagenesis and single-cell high-throughput screening, offering distinct advantages over both conventional mutagenesis and non-droplet-based approaches.

1. High mutation diversity with preserved viability [21]: ARTP mutagenesis technology can induce effective genomic mutations in a large number of microorganisms or cells within a short period. ARTP introduces extensive genetic diversity without harsh chemical agents or thermal damage, preserving the physiological integrity of microbial cells. This ensures that nearly all mutants remain viable for immediate downstream encapsulation and growth within microdroplets—significantly reducing recovery time compared to UV (Ultraviolet, UV) or EMS (Ethyl methane sulfonate, EMS) mutagenesis.
2. Accurate screening and high-throughput detection [22]: In DBMF, each ARTP-derived mutant is compartmentalized in an oil-isolated droplet, eliminating intercellular interference and enabling precise measurement of enzyme activity, metabolic output, or stress resistance. By dispersing microbial cells into individual droplets, each mutant can be isolated for observation and screening. This approach contributes to enhanced screening accuracy and helps mitigate issues such as cross-contamination and misselection, which are commonly encountered in conventional methods, thereby supporting the acquisition of authentic and pure selected strains. Compared with flow cytometry, the droplet approach allows simultaneous assessment of enzyme expression, stability, stereoselectivity, and substrate specificity within a fully customizable microenvironment.
3. Diversity and adaptability: This mutation breeding system can generate high genetic diversity during the mutagenesis process. The various phenotypes produced by random mutations provide researchers with abundant materials to choose from. This allows researchers to more flexibly optimize microorganism performance according to research objectives and practical needs. It also enables the system to adapt to different production conditions and market demands.
4. Adaptive and evolvable feedback loop: The integration of ARTP-induced genetic variability and DBMF-enabled real-time phenotypic analytics forms an adaptive loop: the best-performing mutants identified in each cycle inform subsequent mutagenesis parameters, enabling directed evolution with minimal human intervention. This feedback mechanism transforms mutation breeding from a static process into a self-optimizing system.
5. Minimal Reagent Consumption and Operational Costs: ​By coupling ARTP’s rapid, uniform mutagenesis with DBMF’s parallelized droplet generation (>20,000 s^− 1^), the overall screening speed is increased by 10^4^-10^5^fold compared with traditional microplate assays. Reagent consumption is simultaneously reduced by up to 10^6^-10^7^ times, making large-scale mutant screening economically feasible.
6. Environmental friendliness and safety: Unlike traditional chemical mutagens, ARTP mutagenesis technology uses a physical method. This avoids potential environmental pollution and health risks to operators. The ​​closed nature of the microfluidic system​​ further minimizes aerosol exposure to biological materials, providing an additional layer of containment and safety for the operator.
7. Simplified operational procedures: The integration of ARTP and droplet microfluidic cultivation technologies streamlines the mutation breeding process and reduces experimental complexity. Researchers can utilize automated equipment and data analysis software to achieve efficient screening and cultivation of mutants. This further enhances laboratory productivity and result reliability.

## Applications in biosynthesis and fermentation engineering

High-throughput mutation-breeding systems are increasingly used in biosynthesis and fermentation engineering. They can rapidly generate numerous microbial mutants. By screening suitable mutants, they enhance microbial product synthesis in specific metabolic pathways, increase target metabolite levels, and reduce by-product formation in fermentation. (See Table 1 for applications in biosynthesis and fermentation engineering.)

**Table 1:** Applications of High-Throughput mutagenesis screening systems in biosynthesis and fermentation engineering

Organism	Target Product/Trait	Obtained Phenotype	References
Yarrowia lipolytica	High-yield erythritol mutant	Obtained mutant strain G31 with erythritol titer increased to 220.5 g L^− 1^, productivity enhanced to 1.8 g L^− 1^ h^− 1^, and yield improved to 0.6 g g^− 1^ from crude glycerol	[23]
Bacillus cereus	High-yield chitosanase mutant	Obtained a genetically stable high-yield mutant strain with 3.66-fold increased enzyme activity, significantly improving chitosanase production in B. cereus	[24]
Rhodobacter sphaeroides	High-yield coenzyme Q10 mutant	Obtained mutant strain R.S 17 with 80.37% increased product yield. In fed-batch fermentation coenzyme Q10 concentration and cell density reached 236.7 mg/L and 57.09 g/L respectively	[25]
Rhodococcus equi	High-yield chitin deacetylase (CDA) mutant	Obtained high-yield strain B4 with maximum CDA production 3.15 times higher than parent strain and total enzyme production 3.90 times that of original strain	[26]
Yarrowia lipolytica	High-yield xylanase strain	Final screened strain showed xylanase activity up to 4.7 times higher than wild-type	[27]
Corynebacterium glutamicum	High-yield L-glutamic acid mutant	Obtained mutant strain YAG117 producing 16.3 g/L L-glutamic acid in shake-flask fermentation 13.9% higher than parent strain	[28]
	High-yield L-histidine mutant	Selected resistant mutant Cg-F4 producing (0.561 ± 0.016) g/L L-histidine	[29]
Corynebacterium sp.	High-yield L-glutamine mutant	Obtained mutant strain producing 25.7 ± 2.7 g/L L-glutamine	[30]
Auxenochlorella pyrenoidosa	High-yield amino acid mutan	Obtained mutant strain MMC-8 with total amino acid content increased to 44.35% DW (33.4% enhancement), essential amino acid content reaching 21.25% DW, compared to the original strain A4-1.	[31]
Bacillus siamensis	High-yield macrolactins mutant	Obtained mutant strain IMD4036 with a 3.0-fold increase in macrolactins production compared to the parental strain, and further optimization through ZnSO^4^ (30 mg/L) supplementation elevated the yield to 503 ± 37.6 µg/mL	[32]
Lactobacillus plantarum	High-yield bacteriocin mutant	Obtained high-yield bacteriocin mutant with production efficiency increased from 103.48% to 551%	[33]
Sinorhizobium sp.	High-yield vitamin B_12_ mutant	Obtained high-yield mutant strain BCA-24 with VB_12_ production significantly increased from 65.64 mg/L to 104.54 mg/L compared to wild strain	[34]
Saccharomyces cerevisiae	Inhibitor-tolerant ethanol-producing mutant	Obtained mutant strain M8 with 56.29 g L^− 1^ ethanol titer and 0.49 g g^− 1^ yield (96.67% of theoretical) under lignocellulosic inhibitor stress	[35]
Yarrowia lipolytica	High-yield erythritol mutant	Obtained mutant strain S4-9 with 231.2 g L^− 1^ erythritol in 114 h, showing 16.97% higher yield and 26.09% higher productivity than parent strain	[36]
Saccharomyces cerevisiae	High-protein mutant	Obtained mutant strains HF5 and UD11 with 7.40% and 10.92% increased protein content, and 15.15% and 18.18% enhanced protein yield, respectively	[37]
	Lactic acid-tolerant mutant	Obtained mutant strain NCUF309.5-44 with 93.65% higher OD value, 2.29-fold higher ethanol production, and 60.69% more volatile flavor compounds under 4% lactic acid stress	[38]
	High-yield lycopene mutant	Obtained genetically stable high-yield mutant with maximum lycopene production reaching 8.15 g/L	[39]
	High-yield tyrosine mutant	Mutant strain showed p-coumaric acid (p-CA) production 7.6 times higher than wild-type strain, significantly improving tyrosine synthesis capability in yeast cells	[40]
	Low acetaldehyde-producing mutant	Obtained mutant strain LAL-8a with acetaldehyde production reduced by 88.2% compared to wild-type M14	[41]

### Applications in biosynthesis

####  Excessive enzyme producers

High-throughput mutagenesis screening systems have proven to be an effective approach for enhancing enzyme production and optimizing microbial biocatalysts. By combining random mutagenesis with high-throughput adaptive screening, this system enables the rapid creation and selection of strains with superior enzymatic activity and stability. Li et al. [23] established a biosensor-guided adaptive evolution platform integrating ARTP mutagenesis with the automatic microbial adaptive evolution instrument (EVOL cell) to enhance erythritol biosynthesis in Yarrowia lipolytica. Under optimized ARTP conditions (120 W, 190 s), mutant strains were evolved and screened using a growth-coupled biosensor. The engineered strain G31 exhibited markedly improved enzyme activity and metabolic efficiency, achieving an erythritol titer of 220.5 g L^− 1^, productivity of 1.8 g L^− 1^ h^− 1^, and yield of 0.6 g g^− 1^ crude glycerol in a 5 L bioreactor. In chitosan oligosaccharide production, ARTP has effectively generated Bacillus cereus mutants, improving chitosanase yield and activity. Zhang et al. [24] used ARTP to mutate B. cereus, selecting a high yield chitosanase producing strain with 3.66 times higher enzyme activity, significantly increasing enzyme production while keeping enzyme properties stable. ARTP also enhances CoQ_10_ production in Rhodobacter sphaeroides. CoQ_10_ is a key dietary supplement for antioxidants and bioenergy production. Wang et al. [25] obtained mutant R.S 17 via ARTP mutation and screening, with a product yield increase of 80.37%. In fed-batch fermentation, CoQ_10_ concentration and cell density reached 236.7 mg/L and 57.09 g/L, respectively, with CoQ_10_ content 22.1% higher than the parent strain. ARTP mutation also improves enzyme production in Rhodococcus maris. Ma et al. [26] used ARTP mutation and microbial microdroplet culture system (MMC) technology to select a high-yield chitin deacetylase (CDA)-producing R. maris strain B4. Its maximum CDA production was 3.15 times higher than the original strain, with a total fermentation enzyme production of 3.90 times that of the original strain.

Currently, most enzyme expression uses the cytoplasm of E. coli, requiring cell lysis to assess enzyme activity. However, enzymes must be extracellular or on the cell surface to mix with droplet substrates. To solve this, Thomas et al. [27] used yeast secreted heterologous enzymes to construct an efficient expression system. To obtain high-yield heterologous enzyme-producing yeast strains, they employed microbial droplet cultivation technology. Researchers selected five hydrolytic genes from the yeast genome, constructed five heterologous enzyme-producing strains, and successfully expressed them. They designed a microfluidic droplet cultivation platform for yeast experiments. The microfluidic chip cultured individual yeasts, injected enzyme substrates, and classified droplets based on enzyme activity to obtain target strains, screening 150 strains per hour. This high throughput method saved time and improved heterologous enzyme quality.

#### Excessive amino acid producers

High-throughput mutagenesis screening systems significantly enhance amino acid synthesis in various bacterial strains. Many studies confirm its versatility in improving specific amino acid production, including poly-γ-glutamic acid (PGA), L-serine, L-glutamine, L-histidine, and L-isoleucine. Liang et al. [28] combined mutation and metabolic engineering to optimize L-glutamine production, showing potential in various applications. Deng et al. [29] mutated wild type Corynebacterium glutamicum using ARTP and selected a resistant mutant Cg-F4 via an automated high-throughput microbial microdroplet culture system (MMC) and plate screening. The L-histidine yield reached (0.561 ± 0.016) g/L. Additionally, Lv et al. [30]. generated a Corynebacterium strain producing 25.7 ± 2.7 g/L of L-glutamine using ARTP mutation and high throughput screening.

The combined Atmospheric and Room-Temperature Plasma (ARTP) and Microbial Microdroplet Culture (MMC) breeding system has demonstrated remarkable efficacy in enhancing amino acid synthesis in microalgae, showing broad application potential for increasing the production of various high-value amino acids. Liu et al. [31] employed ARTP mutagenesis integrated with a high-throughput Microbial Microdroplet Culture (MMC) system for the directional breeding of Auxenochlorella pyrenoidosa, successfully obtaining the high-yield amino acid mutant strain MMC-8. This mutant achieved a total amino acid content of 44.35% dry weight (DW), representing a 33.4% increase compared to the original strain A4-1. Its essential amino acid content reached 21.25% DW, and the branched-chain amino acid content exceeded 8% DW (a 39.86% increase). Furthermore, the Essential Amino Acid Index (EAAI) surpassed 0.95, meeting the high-quality protein standard stipulated by the FAO/WHO. This work provides an excellent engineered strain for the efficient biomanufacturing of microalgae-derived amino acids.

#### High-yield antibiotic strains

The mutagenesis breeding system has been extensively utilized in antibiotic production, particularly for enhancing the synthesis of macrolactins, which are macrocyclic polyketide antibiotics exhibiting broad-spectrum bioactivities including antiviral, anticancer, antifungal, and antibacterial properties. ​​Zhang et al. [32] employed an integrated approach combining ​​ARTP​​ mutagenesis with a ​​Microbial Microdroplet Culture System (MMC)​​ for high-throughput screening and adaptive evolution of Bacillus siamensis. This strategy successfully generated a mutant strain, IMD4036, which demonstrated a ​​3.0-fold increase​​ in macrolactins production compared to the parental strain. Further optimization through the addition of ​​ZnSO_4_ (30 mg/L) elevated macrolactins yield to ​​503 ± 37.6 µg/mL​​, representing a ​​30.9% enhancement​​ over unsupplemented conditions. This study highlights the efficacy of ARTP-MMC coupling for rapid development of high-yielding antibiotic-producing strains.

Bacteriocins are ribosomally synthesized bioactive peptides or proteins. In the development of bacteriocins, Chen et al. [33] developed several high-yield bacteriocin producing mutants from Lactiplantibacillus plantarum using ARTP mutation. Production efficiency rose from 103.48% to 551%.

#### High yield vitamin producing strains

Mutagenesis breeding serves as an efficient and convenient approach to improve microbial production of vitamins. Vitamin B_12_ (VB_12_), a vital nutritional factor, is widely used in feed, food, and pharmaceutical industries. As a compound that humans and mammals cannot synthesize endogenously, VB_12_ plays crucial physiological roles, including participation in DNA synthesis and regulation, promotion of red blood cell formation, and facilitation of fat and carbohydrate metabolism.

Ensifer adhaerens (formerly Sinorhizobium adhaerens) is a microorganism recognized for its ability to synthesize VB_12_ and has garnered significant attention in recent years. However, its practical application remains limited due to low production yields. To address this, Wang et al. [34] employed Atmospheric and Room-Temperature Plasma (ARTP) mutagenesis to enhance VB_12_ biosynthesis and investigate the underlying mechanisms. Through multiple rounds of mutagenesis, three high-yield mutant strains BCA-24, BCB-14, and BCC-27 were isolated. Notably, the VB_12_ titer of the best-performing mutant, BCA-24, increased significantly from 65.64 mg/L to 104.54 mg/L. Whole-genome resequencing identified 14 mutated genes, among which seven (atpA, gntR, fusA, cobQ, ribD, cirA, and UP) were functionally validated via overexpression in the wild-type strain. These genes were found to positively influence VB_12_ biosynthesis, providing insights into potential metabolic engineering targets for further strain improvement.

This study demonstrates the effectiveness of ARTP mutagenesis in enhancing VB_12_ production in Ensifer adhaerens, offering a promising strategy for industrial-scale microbial vitamin biosynthesis.

### Applications in fermentation engineering

Utilizing ARTP mutagenesis, either alone or in combination with droplet-based microfluidic technology, has been shown to not only enhance the production of target metabolites during yeast fermentation but also reduce the formation of undesirable by-products, thereby expanding the industrial applicability of yeast strains. Gu et al. [35] integrated ARTP mutagenesis with Microbial Microdroplet Culture (MMC) to improve inhibitor tolerance in Saccharomyces cerevisiae during lignocellulosic ethanol fermentation, obtaining mutant strain M8 with strong stress resistance and achieving 56.29 g L^− 1^ ethanol and 0.49 g g^− 1^ yield (96.67% of theoretical). Similarly, Li et al. [36] combined ARTP mutagenesis with Fluorescence-Activated Droplet Sorting (FADS) in a picodroplet co-culture system to evolve Yarrowia lipolytica for enhanced erythritol biosynthesis. The resulting mutant S4-9 reached 231.2 g L^− 1^ erythritol in 114 h, with yield and productivity increased by 16.97% and 26.09%, respectively, compared with the parent strain. Yang et al. [37] employed ARTP mutagenesis integrated with microfluidic Fluorescence-Activated Droplet Sorting (FADS) using Green Fluorescent Protein (GFP) and Red Fluorescent Protein (RFP), combined with comparative transcriptomic analysis, for mutagenesis and breeding of Saccharomyces cerevisiae, resulting in mutant strains HF5 and UD11 that exhibited significant improvements in protein content (7.40% and 10.92%) and yield (15.15% and 18.18%). Fan et al. [38] integrated ​​ARTP mutagenesis with ​​Microbial Microdroplet Culture System (MMC)​​ high-throughput screening approach to develop a lactic acid-tolerant strain of Saccharomyces cerevisiae. The mutant strain ​​NCUF309.5-44​​ was successfully selected, demonstrating remarkable performance under 4% lactic acid stress: a ​​93.65% increase in OD value​​, a ​​2.29-fold enhancement in ethanol production​​, and a ​​60.69% elevation in total volatile flavor compounds​​ compared to the original strain.

Lycopene, a red carotenoid with significant antioxidant activity, is widely used in the food, cosmetics, and pharmaceutical industries. The production of lycopene in Saccharomyces cerevisiae offers an economical and sustainable approach. Zhou et al. [39] combined ARTP mutagenesis with H_2_O_2_ induction technology to achieve a maximum lycopene yield of 8.15 g/L in a 7 L bioreactor. Cai et al. [40] used ARTP technology to perform random mutagenesis on S. cerevisiae. After two rounds of mutagenesis, five high-yield tyrosine mutant strains were obtained, with the highest p-coumaric acid (p-CA) production being 7.6 times higher than that of the wild-type strain, significantly enhancing the ability of yeast cells to synthesize tyrosine.

Additionally, high-throughput mutagenesis screening systems have been employed to obtain fermentation strains with special robustness and low acetaldehyde production. Liu et al. [41] combined ARTP mutagenesis with a high-throughput screening method using a 4-methylpyrazole and disulfiram coating to obtain a S. cerevisiae mutant strain LAL-8a with reduced acetaldehyde production. The acetaldehyde content of this mutant was reduced by 88.2% compared to the wild-type strain M14.

## Applications of high-throughput mutagenesis and screening systems in the biological feed industry

The fermentation of feed plays a crucial role in enhancing the nutritional value and resilience of livestock. Fermentation optimizes feed nutrition and digestibility. Nevertheless, the efficiency of traditional fermentation processes is closely tied to the microbial strains employed. A mutagenesis and screening system based on atmospheric and room temperature plasma (ARTP) and droplet microfluidic technology can identify and cultivate superior microbial strains, driving the optimization of feed fermentation. (See Table 2 for applications in the biological feed industry.)

**Table 2:** Applications of High-Throughput mutagenesis screening systems in biofeed production

Organism	Target Product/Trait	Obtained Phenotype	References
Clostridium butyricum	High-yield chitosanase mutant Enhanced antibacterial activity and stress resistance	The mutants and parent strain showed good antibacterial activity against 9 common pathogens except for Listeria monocytogenes and Staphylococcus aureus with significant differences in antibacterial activity between strains. Significant differences in heat tolerance at 90 °C for 5 min and bile salt tolerance at 0.4% concentration were observed.	[42]
Bacillus coagulans	High stress-resistant mutants	Obtained three mutants (artp-BC5, artp-BC15, artp-BC28) with significantly higher survival rates (18.2%,19.30% and 18.8% respectively) in pH3.0 MRS medium compared to the parent strain WT-03 (7.15%).	[43]
Lactobacillus plantarum	High antibacterial rate mutant	The mutant strain showed 19.4% increased growth rate and 37.5% improved antibacterial rate.	[44]
Streptomyces albus	High salinomycin-producing mutant	The tetracycline- and chloramphenicol-resistant mutant Tet30Chl25 produced salinomycin at 34,712 mg/L in shake-flask culture exceeding 2.0 times the yield of parent strain S12.	[45]

### Engineering of probiotic strains for animal feed applications

Microbial live-cell preparations serve as functional feed additives that enhance nutrient metabolism, modulate gut microbiota composition, and inhibit exogenous pathogenic infections in livestock. However, their practical application in animal husbandry requires robustness to withstand feed processing and harsh gastrointestinal conditions. Thus, probiotic strains for feed additives must exhibit superior stress resistance and stability. Xie et al. [42] employed ​​Atmospheric and Room-Temperature Plasma (ARTP) mutagenesis​​ to engineer Clostridium butyricum, generating mutant strains with dual improvements: enhanced antibacterial activity against pathogens, and increased tolerance to high temperature and bile salts.

Bacillus coagulans, a spore-forming probiotic combining the advantages of lactic acid bacteria and bacilli, demonstrates exceptional resistance to heat, acid, alkali, and salinity. Its spores survive feed processing and gastrointestinal transit while minimizing contamination risks, making it a preferred choice for feed-grade microbial preparations. Liu et al. [43] optimized ARTP mutagenesis (15 s irradiation, 93.84% lethality) coupled with adaptive evolution to develop acid- and bile salt-resistant B.coagulans mutants. When applied to solid-state fermented soybean meal, these mutants elevated nutritional quality, increasing acid-soluble protein (18.2%), crude protein (12.7%), and total amino acid content (9.8%).

Feed contamination by pathogens like Staphylococcus aureus-a leading foodborne toxin producer-poses significant risks. Lactobacillus plantarum, a fermentative probiotic, naturally produces bacteriocins that inhibit such pathogens. Dong et al. [44] achieved a 19.4% growth rate increase and 37.5% higher antibacterial activity in ARTP-mutated L. plantarum strains, demonstrating their potential as natural biopreservatives.

### Antibiotic alternatives

Salinomycin, widely used for coccidiosis control and poultry growth promotion, saw its production enhanced by Zhang et al. [45] through ARTP and ribosome engineering, creating a Streptomyces griseus breeding process. The mutant Tet30Chl25, resistant to tetracycline and chloramphenicol, produced 34,712 mg/L salinomycin, over 2.0 times the parental strain S12.

While antibiotic additives are crucial in feed industries, their extensive use raises safety concerns like drug-resistant bacteria and antibiotic residues. With rising attention on antibiotic resistance spread, livestock has become a focus, leading to stricter regulations on antibiotic use in feeds. Antimicrobial peptides (AMPs), safer antibiotic alternatives with low resistance risk and easy digestibility, are gaining attention. Recent ARTP assisted mutagenesis has improved AMP production efficiency. Nosiheptide, a safe and effective AMP meeting feed additive requirements is seen as an ideal antibiotic replacement.

## Applications of High-Throughput mutagenesis and screening systems in edible mushroom breeding

High yield, stability, quality, and adaptability are crucial in modern edible mushroom breeding. Reports indicate that using mycelium or protoplasts of edible mushrooms as materials, new superior varieties can be developed via high-throughput mutagenesis screening system. This has become a novel approach in edible mushroom breeding. (See Table 3 for applications in edible mushroom breeding.)

**Table 3:** Applications of High-Throughput mutagenesis screening systems in edible fungus Breeding​

Organism	Target Product/Trait	Obtained Phenotype	References
Ganoderma lucidum	High-yield triterpenoid mutant	Obtained mutant strains YB05 and YB18 with mycelial biomass increased by 26.33% and 17.85%, and triterpenoid production significantly enhanced by 32.10% and 15.72%, respectively	[46]
Phellinus baumii	High-flavonoid mutant strain	Obtained a genetically stable high-yield mutant strain A67 with intracellular flavonoid content increased by 88.24%	[47]
Pleurotus djamor	High-laccase mutant strain	Obtained mutant strain 51 − 4 with laccase production of 494.44 U/L ,86.36% higher than wild strain	[48]
Hericium erinaceus	High-polysaccharide mutant strain	Obtained mutant strains HEB and HEC with polysaccharide content in fermented mycelium increased by 23.25% and 47.45% respectively	[49]
Phellinus baumii	High-flavonoid and polyphenol mutant strain	Obtained mutant strain A67 with flavonoid and polyphenol contents significantly increased by 1.87-fold and 1.33-fold respectively	[50]

The mutagenesis breeding system has been widely applied in research aimed at increasing the production of active components in edible fungi. Feng et al. [46] employed Atmospheric and Room-Temperature Plasma (ARTP) mutagenesis combined with the Microbial Microdroplet Culture (MMC) system for the directional breeding of the Ganoderma lucidum strain G0023. By optimizing MMC parameters (using silicone oil as the oil phase, a cultivation time of ≤ 80 h, and 50 droplets per batch), the mutant strains YB05, YB09, and YB18 were successfully screened. Among these, strain YB18 exhibited a 17.25% increase in mycelial growth rate, while YB05 and YB18 showed biomass increases of 26.33% and 17.85%, respectively. Notably, triterpenoid production was significantly enhanced by 32.10% and 15.72% in YB05 and YB18, respectively.

This system has been used to induce gene mutations and increase flavonoid content in Phellinus baumii. For example, Zhang et al. [47] obtained four high flavonoid yielding mutants of P. baumii through ARTP mutagenesis and screening. Mutant A67 showed the highest intracellular flavonoid production, with an increase of 88.24%.

Beyond high-flavonoid mutants, ARTP mutagenesis has generated specialized strains with improved secondary metabolite biosynthesis. Zhang et al. [48] applied ​​Atmospheric and Room-Temperature Plasma (ARTP)​​ to mutagenize protoplasts of Pleurotus djamor RP. Using guaiacol as a visual screening indicator for laccase-positive phenotypes, they identified 10 promising mutants with elevated laccase activity. The top-performing mutant, 51 − 4, achieved a laccase yield of ​​494.44 U/L​​ in shake-flask culture-an ​​86.36% increase​​ over the wild-type strain. Similarly, Gong et al. [49] demonstrated that ARTP-mutated Hericium erinaceus strains Hericium erinaceus B (HEB) and Hericium erinaceus C (HEC) exhibited ​​23.25% and 47.45% higher polysaccharide content​​, respectively, in fermented mycelia. Notably, strain HEC synthesized a novel β-glucan fraction with a molecular weight of ​ 1.056 × 10^6^ Da​​, absent in the parental strain. Zhang et al. [50] selected wild-type Phellinus baumii SH1 for protoplast preparation and ARTP mutagenesis. From ​​1,139 regenerated colonies​​ displaying phenotypic variations in pigmentation, growth rate, and metabolite profiles, mutant A67 was isolated. This strain showed significant improvements in mycelial biomass (dry weight), total flavonoids (​​1.87-fold increase​​), and polyphenols (​​1.33-fold increase​​) compared to the original strain.

## Applications of High-Throughput mutagenesis and screening systems in environmental remediation

Mutagenesis and screening systems are also widely used in environmental remediation. Using ARTP technology, researchers can identify microorganisms capable of degrading heavy metals or pollutants. These engineered strains can be directly applied for the bioremediation of contaminated soil and water bodies, significantly enhancing environmental remediation efficiency. Moreover, this system demonstrates equal applicability in selecting and breeding biocontrol strains capable of potently inhibiting pathogenic bacteria, thereby indirectly facilitating the restoration and protection of soil ecosystem integrity. By enabling the targeted enhancement of microbial environmental adaptability and functional activity, this platform provides a dual-purpose solution for both pollution treatment and ecological conservation. (See Table 4 for applications in environmental remediation.)

**Table 4:** Applications of High-Throughput mutagenesis screening systems in environmental remediation

Organism	Target Product/Trait	Obtained Phenotype	References
Bacillus amyloliquefaciens	High-yield biosurfactant mutant	Obtained 1 mutant strain that achieved 45.44% petroleum hydrocarbon removal rate in soil column leaching experiments by modifying related enzyme activity	[51]
Bacillus velezensis	Chromium-resistant mutant	The minimum inhibitory concentration of chromium increased from 80 mg/L in the original strain to 400 mg/L in the mutant	[52]
Pseudomonas fluorescens	High-yield extracellular polymeric substances (EPS) mutant	Obtained mutant strain T4-2 with significantly improved EPS production and flocculation activity	[53]
Sphingobacterium multivorum	Highly efficient petroleum hydrocarbon (TPH) degrading strain	The mutant strain achieved a maximum TPH degradation rate of 85.1% representing a 48% improvement compared to the wild-type strain	[54]
Bacillus safensis	Antagonistic mutant against soft rot pathogen Erwinia carotovora	Obtained mutant strain R1-15 with a 42.82% increase in inhibition zone diameter and a 62% enhancement in antibacterial activity against Erwinia carotovora	[55]

Wang et al. [51] used Bacillus amyloliquefaciens A3, a wild type strain producing lipopeptide biosurfactants, to enhance biosurfactant production via ARTP mutagenesis for removing soil petroleum hydrocarbons. In soil column leaching experiments, they achieved a 45.44% removal rate by modifying relevant enzyme activities.

The Bao team [52] found Bacillus velezensis LYB-23, a chromate resistant bacterium, in waste chromium residues. Using a co-cultivation and ARTP based approach, they increased the minimum inhibitory concentration of chromium for B. velezensis from 80 mg/L in the original strain to 400 mg/L in the mutant strain. This novel method significantly improved chromium tolerance and enhanced the bacteria’s ability to reduce and absorb this pollutant, showing great potential for tailoring bacteria for environmental remediation.

Yang et al. [53] isolated Pseudomonas fluorescens from soil, a bacterium that produces extracellular polymeric substances (EPS). Using ARTP technology, they obtained a mutant strain, T4-2, with significantly higher EPS production and improved flocculation activity. The EPS from T4-2 has a high adsorption capacity for chromium and shows great potential for removing cadmium from contaminated soils and lake waters, offering a promising solution for environmental remediation.

Du et al. [54] employed bio-remediation technology to isolate total petroleum hydrocarbon (TPH) degrading bacteria from oilfield produced water through enrichment culture, primary, and secondary screening. Using a combination of UV and plasma mutagenesis, they used 96-well plate fermentation instead of shake flask fermentation and a high-throughput screening method based on a multi-functional microplate reader with dual-wavelength UV spectroscopy to select efficient TPH-degrading strains. The degradation rate reached up to 85.1%, an 85.1% improvement over the wild type strain. This approach significantly reduces TPH levels in the environment and effectively remediates crude oil contaminated sites.

Wei et al. [55] utilized ARTP mutagenesis combined with a ​​Droplet Entrapping Microfluidic Cell-sorter (DREM Cell)​​ high-throughput screening system to optimize the selection of a Bacillus safensismutant strain R1-15 from a soil microbial community. The mutant exhibited a ​​42.82% increase in inhibition zone diameter​​ against Erwinia carotovorapathogens compared to the wild-type strain, along with a ​​62% enhancement in antibacterial activity​​ in sterile deep-well plate fermentation broth, while maintaining excellent genetic stability. This mutant demonstrated high efficiency in suppressing soft rot Erwiniapathogens, reducing crop disease incidence, decreasing reliance on chemical pesticides, and promoting soil microecological health. This study provides an efficient biocontrol platform for agricultural pollution management and sustainable ecosystem governance.

## Future perspectives

The high-throughput mutagenesis and screening system based on atmospheric and room temperature plasma (ARTP) mutagenesis and droplet microfluidic technologies demonstrates significant potential in the field of microbial breeding. However, research in this area remains at an early stage, with a limited number of studies achieving deep integration of ARTP and DBMF. Most current studies employ ARTP or DBMF separately, and only a few have realized iterative coupling within a closed-loop evolutionary framework. Furthermore, challenges persist regarding system scalability, long-term genetic stability, robustness of process parameters, and industrial implementation. Despite these limitations, the integrated strategy offers distinct advantages in accelerating random mutagenesis, enabling single-cell precision screening, and enhancing evolutionary efficiency. As relevant technologies continue to advance, this system is expected to find broader applications, thereby increasing its practical utility.

## Data Availability

No datasets were generated or analysed during the current study.

## Abbreviations

ARTP: Atmospheric and room-temperature plasma
DBMF: Droplet-based microfluidic
MMC: Microbial Microdroplet Culture system
CDA: Chitin deacetylase
PGA: poly-γ-glutamic acid
VB_12_: Vitamin B_12_
p-CA: p-coumaric acid
AMP: Antimicrobial peptides
HEB: Hericium erinaceus B
HEC: Hericium erinaceus C
EPS: Extracellular polymeric substances
TPH: Total petroleum hydrocarbon
UV: Ultraviolet
EMS: Ethyl methane sulfonate
EVOL cell: Automatic microbial adaptive evolution instrument
DW: Dry weight
EAAI: Essential Amino Acid Index
FADS: Fluorescence-Activated Droplet Sorting
GFP: Green Fluorescent Protein
RFP: Red Fluorescent Protein
DREM Cell: Droplet Entrapping Microfluidic Cell
